# Anteflexion of the Uterus and Its Associated Pathological Conditions—Their Prevention and Treatment

**Published:** 1885-07-20

**Authors:** G. G. Roy

**Affiliations:** Atlanta, Ga.; Professor of Materia Medica, etc., Southern Medical College


### Southern Medical Record.

EDITORS:
THOMAS S. POWELL, M. D. R. C. WORD, M. D.

*R. C. WORD, M.D., Managing Editor.*

☞ All Communications and Letters on Business connected with the RECORD must be addressed to the Managing Editor.

VOL. XV. ATLANTA, GA., JULY 20, 1885. No. 7.

### ORIGINAL AND SELECTED ARTICLES.

### ANTEFLEXION OF THE UTERUS AND ITS ASSOCIATED PATHOLOIGCAL CONDITIONS—THEIR PREVENTION AND TREATMENT.

BY G. G. ROY, M.D., ATLANTA, GA.
Professor of Materia Medica, etc., Southern Medical College.

In the American Journal of Obstetrics for September, 1883, and December, 1884, appears a well prepared and interesting article from W. Gill Wylie, M.D., Professor of Gynecology in the New York Polyclinic, and Gynecologist to Bellevue Hospital, N. Y.

In the first part of this paper Dr. W. undertakes to show the following : "The normal uterus is flexed forward, and so placed in the pelvis that downard pressure does not act directly on it.

It is so suspended, surrounded and sustained by an elastic medium,the pressure of which is so regulated by the heart's action and the contractility and elasticity of the muscles and fasciæ of the blood vessels and the abnormal walls—the vital pressure—that the weight of the normal-sized fundus has but a slight tendency to increase the anteflexion ; the body of the uterus being sustained and protected as though it were surrounded by water."

He thinks that "many authors have overlooked these facts, and have been led too lay to much stress upon the effects of blows, falls, etc., in producing anteflexion, and have taught that bending closes the uterine canal mechanically and thereby creates the symptoms, etc."

Agreeing in the main with the conclusions of the writer as stated, I cannot hold that "authors have placed too much stress upon the *effects* of falls, blows, etc., in producing anteflexion."

Believing that the "vital pressure" is a great conservator of the uterus from the *direct* injury from falls, blows, etc., still I must believe that they are *indirectly* the cause of a majority of the anteflexions of the young, before the age of puberty.

Dr. W. very properly says, that, "anteflexion does not constitute the disease, but is the result of imperfect development or of disease—a condition associated with certain pathological changes in the tissues of the organs.

He enumerates these pathological conditions, viz.: "1st. Congenital influences. 2nd. General and special causes tending to prevent perfect development, and thereby producing premature atrophy and degeneration. 3rd. Diseases which soften the walls or those which enlarge the fundus, or lengthen the cervix, or relax or overstretch the suspending ligaments, etc., etc."

I believe that congenital influences, so often produce imperfect development or disease of the uterus in the young, and this is likely to result in anteflexion, but from my observation, the most frequent cause is found under the 2nd head, viz.: "General and special causes tending to prevent perfect development, and thereby inducing premature atrophy and degeneration."

Now, the most common and prominent *special cause*, in my judgment, is injury to the nervous centres controlling the nutrition and health of the uterus, by falls or blows received during childhood, and this produces the imperfect development or disease resulting in the anteflexion.

I do not believe that the mechanical effect of the flexion *always* causes dysmenorrhœa, etc., but that these symptoms may be due to the degenerate, hyperesthetic and more or less contracted or "stenosed condition of the tissues, chiefly at or about the os internum." That this is the location of the obstruction, I have no doubt.

My observations lead me to believe that anteflexion is *the displacement* of the young, unmarried woman, and of those who do not bear children after the usual period when married, and that in the majority of these cases will be found imperfect development of the organ with "a stenosed condition of the tissues, chiefly at or about the os internum," and the causes, from a correct history of the subject, may be traced to injury during girlhood, from falls or blows upon the back, producing more or less concussion of the spine with its attendant results, which at the time was not severe enough to arouse the attention or anxieties of the family or friends, and the symytoms resulting therefrom, though accompanied with very severe pain in the back, and pain with soreness through the pelvic region. The fact that they may be connected with the uterus and itsappendages, has never suggested itself to any one until a vaginal examination reveals the anteflexion of an imperfectly developed organ for the first time when obstructive dysmenorrhœa demands a physician's examination.

When a girl under puberty receives an injury from a severe fall in horseback riding (falling backwards a common cause), or is thrown violently from a vehicle in rapid motion, resulting in a more or less degree of concussion of the spine, and probably lifted up insensible, every organ of the body is interrogated for the symptoms of the apparent injury *but the womb and its appendages*, when, in fact, many of the obscure and obstinate symptoms attending the case are due to injury of these, retarding if not arresting their development, and resulting in the anteflexion which the gynecologist is left to discover at his first examination. The eyes of the patient and her friends being opened for the first time, their memory will carry them back to the date of the injury, and probably the beginning of more or less impairment of the child's health.

My experience is, that girls thus suffering are especially liable to attacks of acute endo-metritis from very slight causes, such as getting the feet damp when menstruating, or otherwise becoming chilled in any part of the body. I agree with Prof. W., "that faulty general development of the body in childhood, from bad food, air and other unhygienic surroundings, together with lacing, etc.," are all important causes as tending to impair the natural development of the organ, and are potent factors of the conditions under consideration.

The doctor's plan of treatment is good, especially do I endorse the constitutional treatment, as an invaluable auxillary, and often a prime necessity for the relief of these cases.

While endorsing in the main the treatment of Prof. W., I desire to call attention to the very excellent results in my hands, in the use of seatangle (lammaria) tents in overcoming the atresia of the cervical canal, at and oftentimes anterior to the os internum as usually found in anteflexions in young women.

After the necessary depletion of the uterus by means of tampons of absorbent cotton, alterative application of iodoform, or tr. iodine, carbolic acid and glycerin, I begin dilitation of the cervical canal in the following manner:

If the canal will admit the smallest size tent, bent at the angle the flexion may have, I introduce it half an inch beyond the flexion (and that I have not found attended with much pain or inconvenience) and let it remain until *fully expanded*, and then withdraw it.

If the womb has not become too sensitive from this procedure, another tent a size larger is introduced and dealt with in the same way, and this is repeated till the canal is as large as desirable. Now when the seatangle tent is bent (and when of good quality and flexible texture, without blemishes, this may be done, to almost an acute angle) and introduced, as it expands it straightens, and in doing so, diminishes the flexion to a certain degree also. Now, when the womb is not held by adhesion or other causes, this gradual dilitation with the tents will often overcome the flexion entirely without the loss of blood, and with no more pain than attends the ordinary use of these tents.

Sometimes we meet with cases in which there is complete occlusion of the internal os, together with a considerable portion of the canal anterior to the os, the result of organized plastic exudation, and through which we can neither introduce the tents or the dilators without using an unjustifiable degree of force. In such cases I have had excellent results in opening up a passage through the canal by using sea tangle tents in the following manner:

First, measure the length of the cervical canal, unobstructed, and cut the tent this length, and introduce it well up (firmly) against the lower point of obstruction and retain it there by compresses of absorbent cotton nicely and evenly adjusted till the vagina is well filled and the womb is securely steadied. This tent is allowed to remain until it gives its utmost degree of expansion and then withdrawn. You will then discover that a perceptible degree of this obstruction has been overcome, and quite an increased length of the canal gained. Another tent of sufficient length to meet this gain, is introduced and retained in the same way; and this plan is continued (using a longer tent each time) until the occlusion of the canal is overcome and the internal os is sufficiently open to admit a tent, bent at the angle of flexion then remaining, and it will be discovered that a tent several sizes larger than the one used at the beginning of the treatment may now be passed. After this tent has served its purpose, it is withdrawn and another, larger, and bent at a more obtuse angle, is introduced, and this plan is continued until full dilitation is effected, when the flexion will be found almost, if not entirely restored.

Without wishing to be tedious to the readers of the RECORD, I will report only a few cases of quite a number successfully treated by this plan :

CASE I.

About five years ago Miss C., aged 20, medium size, dark hair and complexion, was brought to this city from a distant portion of the State, an emaciated and suffering invalid, unable to walk, with an irritable bladder, constipation and extreme nervousness. Being a member of a wealthy family, no expense had been spared in consulting physicians and in trying the virtues of the different health resorts of the South; she continued to grow worse, and when she reached this city her condition was a very sad one.

There had been a complete suppression of the menstrual functions for over a year. A digital examination revealed an anteflexed and an insufficiently developed uterus. An examination with the sound revealed a completely occluded cervical canal from and including the os internum to within one-eighth of an inch of the os externum : the occlusion was so complete that a small filiform elastic bougie could not be introduced. I introduced into the free portion of the canal a piece of lammaria tent as long and as large as would be admitted, and secured it with pledgets of absorbent cotton as heretofore described in this article. With the expansion of this I gained a little in size and length of the canal. This process was repeated every twenty-four hours with longer and larger pieces of the tent through the os internum. The tents were now continued every two or three days until the internal os was fully and completely dilated and the anteflexion entirely overcome.

In conjunction with this local treatment, active measures for the restoration of her general health were used with the most gratifying results.

The young lady was able in a few months to walk a mile or two in the city without fatigue or discomfort, her general health greatly improved—the gain in flesh and strength to a degree hardly to be expected when we take into consideration the fact that there was apparently complete atrophy of the ovaries, and they did not respond to any of the ordinary remedies for the restoration of the catamenia, including a persistent use of electricity.

In eliciting the history of this case, I learned that, at the age of fourteen or fifteen, she was thrown from a buggy, just before a menstrual period, and struck violently upon the back, producing concussion of the spine. When the "period" came, it was painful and scant; and she was compelled to take her bed, to which she was confined several months, with great pain through the pelvic region, in the back, and lower part of the abdomen. Each recurring "period" was more painful and scant, until menstruation ceased entirely, followed by an aggravation of all these painful symptoms. Her general health began rapidly to fail, and, for nearly two years, she had spent most of the time in bed, a wretched, nervous, and, to herself and friends, a hopeless invalid, whom Death would soon claim as its victim.

### CASE II.

Miss E. I. S., age twenty ; tall, slenderly built ; had always enjoyed good health ; one of a large family of daughters, all of whom are healthy ; parents healthy. About two years previous to her consulting me, in 1882, about the onset of a menstruation, she was overtaken, some 20 miles from home, whilst returning in an open buggy, by a drenching rain, which continued through the entire trip. A scanty flow appeared that night, accompanied with much pain and soreness of the ovaries and uterus. By morning the flow had ceased, but she felt no serious inconvenience, except the soreness through the pelvis and a general malaise and stiffness of the body, from her five hours' trip in a cold, drenching rain. Three or four days after this, she says, while writing a letter, her pen suddenly dropped from her fingers, and she felt as if she would topple over. This sensation was quickly *followed* by a severe pain in the crown of her head, which she thought "must kill her." She endeavored to walk, but was unable from "stiffness of one side." She was put to bed, but could get no relief from this torturing headache until her usual period of menstruation had fully passed. Each menstrual period after this, for many months, was extremely painful, with little or no flow, and invariably attended with this agonizing cephalalgia. After nearly two years of such suffering, having passed through the hands of many physicians, and several "quacks," she was brought to this city on a cot, being too feeble and emaciated to walk.

Upon examination, she was found to be in pretty much the condition of the patient in Case I. There was anteflexion, atrophy or want of natural development of the uterus, and an occlusion from organized lymph (plastic exudation), including the internal os, to within an eighth of an inch of the os externum.

The same mode of treatment as in Case I was adopted with the most gratifying results. In four months the flexion was entirely overcome ; menstruation restored, easy and natural, and she returned home in excellent health, and has since become pregnant ; but not expecting such a thing, by imprudence—ignorantly—a miscarriage was induced.

### CASE III.

Miss E. M., aged twenty, with a previous history of good health, in returning from church, while menstruating, got her feet wet. This was followed by a severe attack of metritis. One month afterwards I was called in consultation, and, upon examination, found a completely anteflexed womb, poor development, and extreme sensitivenss of the internal os. There was no organized occlusion, but the sensitiveness was so acute and intense that she could not allow me to use the tents sufficiently to test their value. Only one, I think, was used. This acute attack of metritis was followed by the most intense, protracted, and intractable case of hysteria it has ever been my misfortune to encounter. Without suffering any pain, even at the subsequent menstrual periods, she brought herself to believe that the fundus or "top of her womb had grown to the front walls of her abdomen," and that if she straightened herself in bed, or assumed the upright posture to walk, it would break away from its moorings, and she would suddenly die ; she, consequently, remained in bed for nearly twelve months, fearing to make an effort to get up, for the reason given.

### CASE IV.

This is a colored cook, aged about twenty-seven, having the most rigidly anteflexed womb I ever met with. Menstruation had become so painful and protracted that for one-third of each month she was unable to perform her daily duties ; in fact, was confined to her bed. After the neck of the womb had been softened (and this should always be done before using the tents), I succeeded in introducing the smallest lammaria tent I could find, bent at almost a right angle. After its expansion, the canal was dilated sufficiently to receive one of larger size ; and this process was continued until the cervical canal was so enlarged, and the anteflexion overcome, as to allow free and painless menstruation—an event hitherto unknown to her.

This woman had suffered so much and so long from dysmenorrhœa that each recurrence brought on violent spasms, for the relief of which I was consulted.

I could mention quite a number of other cases similarly treated, in all of which the undeveloped feature of the womb was present, but I have already consumed more space than I intended.

---

PURE aconitin has no color reaction. It makes, even with concentrated sulphuric acid, an absolutely colorless solution, to which a concentrated solution of sugar may be added without producing the slightest shade of color.

